# Insulin-like growth factor 2 mRNA binding protein 2 regulates proliferation, migration, and angiogenesis of keratinocytes by modulating heparanase stability

**DOI:** 10.1080/21655979.2021.2002495

**Published:** 2021-12-07

**Authors:** Shaomin Zhi, Jun Li, Xiao Kong, Xuemei Xie, Qiangli Zhang, Guoxiang Fang

**Affiliations:** Department of Emergency, Xi’an No. 3 Hospital, Xi’an, Shaanxi, P.R. China

**Keywords:** Wound healing, keratinocyte, IGF2BP2, HPSE, proliferation, migration, angiogenesis

## Abstract

Wound healing is related to proliferation, migration, and angiogenesis of keratinocytes. Insulin-like growth factor 2 mRNA binding protein 2 (IGF2BP2) is an important N6-methyladenosine (m6A) reader, which is involved in multiple processes, including wound healing. However, the function and mechanism of IGF2BP2 in keratinocyte processes are largely uncertain. In the present study, expression levels of IGF2BP2 and heparanase (HPSE) were detected by quantitative reverse transcription polymerase chain reaction and western blotting assays. Cell proliferation was investigated by cell counting kit-8 (CCK-8) analysis. Cell migration was determined through wound healing assay. Angiogenesis was measured by tube formation assay and vascular endothelial growth factor (VEGF) level using enzyme linked immunosorbent assay (ELISA). The interaction between IGF2BP2 and HPSE was analyzed by RNA immunoprecipitation, pull-down and luciferase reporter analyses. The results showed that IGF2BP2 expression was enhanced in wound healing. IGF2BP2 downregulation constrained HaCaT cell proliferation, migration, and angiogenesis. IGF2BP2 knockdown decreased HPSE expression. IGF2BP2 could regulate HPSE stability by binding with 3ʹ untranslated region (UTR) of HPSE. HPSE upregulation attenuated silencing IGF2BP2-mediated suppression of proliferation, migration, and angiogenesis. As a conclusion, IGF2BP2 knockdown repressed proliferation, migration, and angiogenesis of HaCaT cells by decreasing HPSE stability.

## Introduction

Wound healing is a complex process after the chronic wounds mainly induced by diabetes, vascular disease, and aging [1]. Skin is the largest organ in human body, and keratinocytes are the major cells involved in the wound healing process when the skin is wounded [2]. Keratinocyte proliferation, migration, and angiogenesis are associated with skin wound healing [3–5]. Thus, exploring the potential molecular targets of keratinocyte processes may help to understand the mechanism of wound healing.

N6-methyladenosine (m6A) is the most prevalent mRNA modification, which has important roles in biological functions [6]. Insulin-like growth factor 2 mRNA binding protein 2 (IGF2BP2) is an m6A reader gene that participates in various biological processes in human diseases [7]. IGF2BP2 is reported to facilitate cell proliferation, migration, and angiogenesis in human cancer cells, like colorectal cancer and lung cancer cell lines [8,9]. A previous study reports that IGF2BP2 contributes to keratinocyte migration in skin wound healing [10]. However, the roles of IGF2BP2 in keratinocyte proliferation and angiogenesis are largely unknown, and the related mechanism remains limitedly understood.

Heparanase (HPSE) is a primary enzyme related to the breakdown of glycosaminoglycan heparan sulfate [11], and it is linked to the wound healing processes [12]. Previous study showed that HPSE contributes to cell proliferation, migration, and angiogenesis in cervical cancer and pancreatic cancer cells [13,14]. However, the role and mechanism of HPSE in keratinocyte processes during wound healing are mostly uncertain.

Here, we hypothesized that IGF2BP2 regulated HPSE stability to modulate keratinocyte processes. The purpose of our research was to analyze the role and mechanism of IGF2BP2 and HPSE in keratinocyte proliferation, migration, and angiogenesis, which may indicate novel target for treatment of impaired wound healing.

## Materials and methods

### Bioinformatics analysis

The differentially expressed genes in wounds on day 7 and 14 after wounding were predicted using Gene Expression Omnibus (GEO) database (http://www.ncbi.nlm.nih.gov/geo/), based on the GSE113081 dataset, which showed the differential genes in dermal wound healing of mice, with a cutoff at *p* < 0.05, log fold change (FC) > 1 or < −1 [15]. The targets of IGF2BP2 were predicted using starBase (http://starbase.sysu.edu.cn/index.php), a platform for RNA–RNA interaction [16]. The overlapping targets were screened using jvenn tool (http://bioinfo.genotoul.fr/jvenn) [17]. The potential pathway was analyzed via Gene Ontology (GO) and Kyoto Encyclopedia of Genes and Genomes (KEGG) enrichment analyses via KOBAS (http://kobas.cbi.pku.edu.cn/kobas3) [18].

### Cell culture and transfection

Human keratinocytes (HaCaT cells) were provided by CLS Cell Lines Service (cat. no. 300,493; Eppelheim, Germany), and incubated in Dulbecco’s Modified Eagle Medium (DMEM) (Gibco, Grand Island, NY, USA) supplemented with 10% fetal bovine serum (Gibco), 4.5 g/L glucose (Sigma-Aldrich, St. Louis, MO, USA), 2 mM L-glutamine (Sigma-Aldrich) and 1% penicillin/streptomycin (Gibco) at 37°C in 5% CO_2_. Human vascular endothelial cell line HUVEC cells were provided by Procell (cat. no. CL-0122; Wuhan, China), and cultured in DMEM medium (Gibco) plus 10% fetal bovine serum, 0.1 mg/mL heparin (Sigma-Aldrich), 30 μg/mL endothelial cell growth supplement (Sigma-Aldrich), and 1% penicillin/streptomycin at 37°C in 5% CO_2_.

The pcDNA3.1-based IGF2BP2 or HPSE overexpression vectors (pcDNA-IGF2BP2 or pcDNA-HPSE) was generated via inserting the sequence of IGF2BP2 or HPSE in the vector, and the empty vector (Thermo Fisher Scientific, Waltham, MA, USA) functioned as negative control (pcDNA3.1). Adenovirus expressing shRNA to IGF2BP2 (sh-IGF2BP2) and negative control (sh-NC) were generated by Vigene Biosciences, Inc. (Shandong, China). A total of the sequences for sh-IGF2BP2 were shown as below: sh-IGF2BP2#1: AGTGAAGCTGGAAGCGCATATTTCAAGAGAATATGCGCTTCCAGCTTCACTTTTTTT; sh-IGF2BP2#2: TTCCCGCATCATCACTCTTATTTCAAGAGAATAAGAGTGATGATGCGGGAATTTTTT; sh-IGF2BP2#3: ATCAAACAGCTGGCGAGATTCTTCAAGAGAGAATCTCGCCAGCTGTTTGATTTTTTT; sh-IGF2BP2#4: AGCGCAAGATCAGGGAAATTGTTCAAGAGACAATTTCCCTGATCTTGCGCTTTTTTT). HaCaT cells were transfected with 1 μg vectors using Lipofectamine 3000 (Thermo Fisher Scientific) according to the protocols as reported previously [19]. After post-transfection for 24 h, cells were collected for western blotting, qRT-PCR or further functional analyses.

### Western blotting

Protein levels were detected by western blotting referring to the protocols in a previous report [20]. In brief, HaCaT cells were lysed in radio-immunoprecipitation assay buffer (Beyotime, Shanghai, China), and extracted protein was quantified by bicinchoninic acid assay kit (Thermo Fisher Scientific). Protein samples (20 μg) were subjected to sodium dodecyl sulfate-polyacrylamide gel electrophoresis, and transfer of polyvinylidene fluoride membranes (Thermo Fisher Scientific). After incubating in 5% fat-free milk, the membranes were incubated with primary antibodies for IGF2BP2 (ab129071, 1:3000 dilution, Abcam, Cambridge, UK), HPSE (ab254254, 1:3000 dilution, Abcam), or β-actin (ab8227, 1:3000 dilution, Abcam) overnight, and then incubated with horseradish peroxidase-conjugated IgG (ab205718, 1:8000 dilution, Abcam) for 2 h, followed by exposure to enhanced chemiluminescence kit (Beyotime). The visualized blots were analyzed via Image J software (NIH, Bethesda, MD, USA), with β-actin as a reference.

### Cell counting kit-8 (CCK-8)

Cell proliferation was measured using CCK-8 according to a previous study [21]. In brief, HaCaT cells (2 × 10^4^) were added into 96-well plates. After grown for 0, 24, 48, 72 h, 10 μL CCK-8 (Beyotime) was infused, and cells were cultured for 2 h. The optical density value at 450 nm was detected with Epoch2 microplate reader (Biotek, Winooski, VT, USA).

### Wound healing analysis

Cell migration was investigated by wound healing assay referring to protocols as reported previously [19]. In brief, HaCaT cells (2 × 10^5^) were dispersed in 6-well plates, and grown to 90% confluences. A pipette tip was used to make a wound. Cells were incubated for another 24 h. Wounds were imaged under a microscope (Olympus, Tokyo, Japan) at 0 and 24 h. Relative migratory rate was calculated according to the wound closure, and normalized to the control group.

### Enzyme linked immunosorbent assay (ELISA)

HaCaT cells (5 × 10^4^/well) were seeded in 24-well plates, and cultured for 48 h. Culture supernatants were collected, and utilized for measuring vascular endothelial growth factor (VEGF) level using Human VEGF ELISA kit (R&D Systems, Minneapolis, MN, USA) in line with the manufacturer’s protocols [22]. The absorbance was detected through an Epoch2 microplate reader at 450 nm. The secretion level of VEGF was calculated based on the standard curve.

### Tube formation analysis

Angiogenesis was investigated by tube formation analysis according to a previous report [23]. In brief, HUVEC cells (1 × 10^4^/well) were added in Matrigel-coated 96-well plates overnight. The medium was changed to the medium of HaCaT cells with indicated transfection for 24 h. The tube formation was imaged under a microscope.

### Quantitative reverse transcription polymerase chain reaction (qRT-PCR)

RNA levels were measured by qRT-PCR according to the protocols as previous report [20]. HaCaT cells were lysed in Trizol (Thermo Fisher Scientific) for mRNA isolation. 800 ng RNA was reversely transcribed through an M-MLV reverse transcription kit (Promega, Madison, WI, USA), and then mixed with SYBR (TaKaRa, Otsu, Japan) and primer pairs (Genscript, Nanjing, China) for qRT-PCR. The specific primer pairs were shown as follows: IGF2BP2 (sense: 5ʹ-AGCCTGTCACCATCCATGC-3ʹ; antisense: 5ʹ-CTTCGGCTAGTTTGGTCTCATC-3ʹ), HPSE (sense: 5ʹ- GCACAAACACTGACAATCCAAG-3ʹ; antisense: 5ʹ-AAAAGGATAGGGTAACCGCAA-3ʹ), β-actin (sense, 5ʹ-CTTCGCGGGCGACGAT-3ʹ; antisense, 5ʹ-CCACATAGGAATCCTTCTGACC-3ʹ). Relative mRNA level of IGF2BP2 or HPSE was calculated with β-actin as control referring to 2^−ΔΔCt^ method [24].

### Actinomycin D assay

The stability of HPSE was analyzed using Actinomycin D according to a previous report with some modifications [25]. In brief, HaCaT cells transfected with sh-NC or sh-IGF2BP2 were treated with 2 μg/mL Actinomycin D (Sigma-Aldrich) for 0, 1, 2, or 4 h. Then, total RNA was isolated, and HPSE mRNA level was examined using qRT-PCR. Relative mRNA remaining level was exhibited as percentage of the non-treated group.

### RNA immunoprecipitation (RIP) analysis

The interaction between IGF2BP2 and HPSE was analyzed by RIP assay following a previous report with some modifications [25]. RIP analysis was performed with a Magna RIP kit (Sigma-Aldrich). In brief, 1 × 10^7^ HaCaT cells were lysed in RIP lysis buffer, and incubated with the magnetic beads coated with IGF2BP2-specific antibodies (ab117809, 1:200 dilution, Abcam) for 6 h. IgG served as negative control. Coprecipitated RNAs were isolated, and HPSE enrichment level was detected.

### Pull-down analysis

RNA pull-down analysis was conducted through Magnetic RNA-Protein pull-down kit (Thermo Fisher Scientific) according to a previous report with some modifications [25]. The biotinylated wild-type (WT) or mutant (MUT) HPSE probe was synthesized via RiboBio (Guangzhou, China). 1 × 10^7^ HaCaT cells were lysed using RIP lysis buffer, and incubated with the synthesized probes and streptavidin-coated magnetic beads overnight. Enriched IGF2BP2 was analyzed by Western blotting. Input represented the positive control.

### Luciferase reporter analysis

The interaction between IGF2BP2 and HPSE was analyzed by luciferase reporter analysis referring to a previous report with some modifications [23]. In brief, the WT or MUT sequence of HPSE 3ʹ untranslated region (UTR) with IGF2BP2 binding sites was inserted in the psi-CHECK2 vector (Promega), generating the WT and MUT HPSE luciferase reporter vectors. These luciferase reporter vectors were co-transfected with pcDNA3.1 or pcDNA-IGF2BP2 in HaCaT cells. After 24 h, the luciferase activity was detected utilizing dual-luciferase assay kit (Promega).

### Statistical analysis

All cellular experiments were conducted 3 times with three replicates. Results were exhibited as mean ± standard deviation. The difference was investigated using Student’s t-test or one-way analysis of variance followed by Tukey post hoc test via GraphPad Prism 8 (GraphPad, La Jolla, CA, USA). *p* < 0.05 indicates the difference was significant.

## Results

### The analysis of differentially expressed genes in wound healing

**Figure 1.**
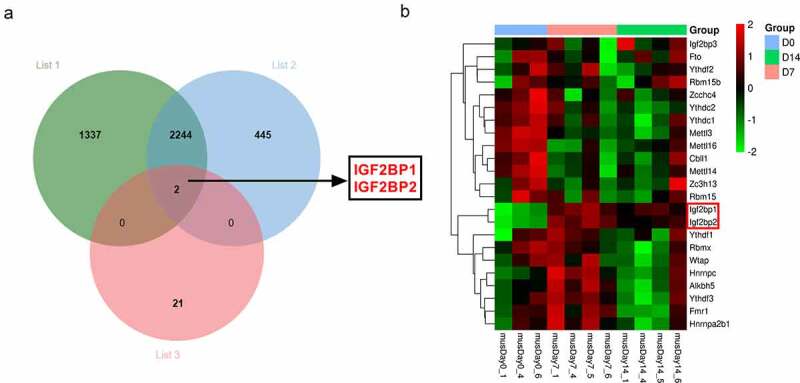
The differentially expressed m6A-related genes in wound healing. (a) The overlapping targets of differentially expressed genes at day 7 (list 1), or day 14 (list 2) from GSE113081 dataset, and 23 m6A-related genes. (b) The heat-map of 23 m6A-related gene levels from GSE113081 dataset

The m6A-related genes have been elucidated in the processes of wound healing. The purpose of this work is to explore what role m6A reader IGF2BP2 plays and how it works in the processes of wound healing. IGF2BP2 was hypothesized to regulate the stability of HPSE in wound healing. To explore the potential m6A-related genes involved in wound healing, the differentially expressed genes in wounds on day 7 and 14 after wounding according to the GSE113081 dataset (Supplementary Table 1). There are 3583 differentially expressed genes (list 1) in wounds on day 7 vs 0 (*p* < 0.05, logFC > 1 or < −1), and 2691 differentially expressed genes (list 2) on day 14 vs 0 (*p* < 0.05, logFC > 1 or < −1). By matching the 23 m6A-related genes and the two groups of differentially expressed genes in wounds, only two genes (IGF2BP1 and IGF2BP2) were overlapped (Figure 1(a)). Moreover, the expression levels of the 23 m6A-related genes in excisional wounds were analyzed based on the GSE113081 dataset. The results of heat-map showed IGF2BP1 and IGF2BP2 were upregulated most in the wounds on day 7 and 14 (Figure 1(b), Table 1). Hence, we hypothesized IGF2BP1 and IGF2BP2 might have important roles in wound healing. IGF2BP2 (*p = *2.86E-08, or 8.03E-07, respectively) with higher statistical significance than IGF2BP1 (*p = *3.18E-04, or 1.48E-03, respectively) was selected for further experiments.

**Table 1.** The expression levels of the 23 m6A-related genes in excisional wounds were analyzed based on the GSE113081 dataset

Gene Name	0d vs 7d		0d vs 14d	
	logFC	P.Value	logFC	P.Value
METTL3	−0.78383	0.0000221	−0.77178	0.0000291
METTL14	−0.76027	0.0001825	−0.42714	0.0080253
METTL16	−0.69565	0.0001576	−0.33093	0.0182622
WTAP	0.066479	0.2866168	−0.04842	0.4342335
RBM15	−0.10641	0.2093974	−0.02527	0.7575772
RBM15B	−0.00079	0.9949037	0.143624	0.2608793
ZCCHC4	−0.50811	0.0157045	−0.29808	0.1147373
ZC3H13	−0.202	0.0320745	0.016905	0.8397182
CBLL1	−0.42241	0.0011503	−0.19746	0.0628636
FTO	−0.14618	0.2145056	0.099104	0.3875844
ALKBH5	0.339025	0.0002211	0.026796	0.6769276
YTHDF1	0.164353	0.1925625	0.141789	0.2595404
YTHDF2	−0.039	0.7121079	−0.00102	0.9922799
YTHDF3	0.222981	0.0130163	−0.05572	0.4763042
YTHDC1	−0.31508	0.0027614	−0.32962	0.0021302
YTHDC2	−0.2282	0.0254493	−0.25467	0.0157144
HNRNPC	0.46445	0.0000661	0.14834	0.0753209
HNRNPA2B1	0.265779	0.0063612	−0.04754	0.5622637
FMR1	0.284393	0.0281284	−0.14158	0.243652
IGF2BP1	4.912674	0.000318	4.095357	0.0014851
IGF2BP2	2.611768	2.86E-08	1.933357	8.03E-07
IGF2BP3	−0.02715	0.8859068	0.351222	0.0748101
RBMX	−0.07801	0.4464025	−0.31555	0.0098493

### IGF2BP2 knockdown reduces cell proliferation, migration, and angiogenesis

**Figure 2.**
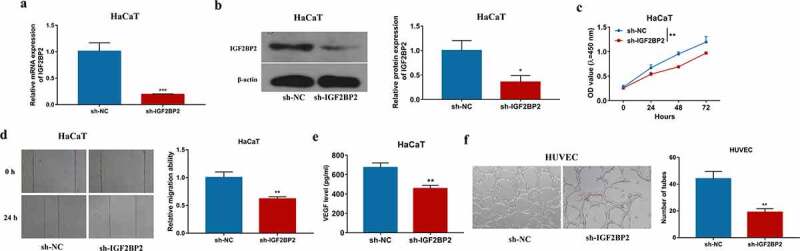
The influence of IGF2BP2 on HaCaT cell proliferation, migration and angiogenesis. (a and b) qRT-PCR and western blotting for IGF2BP2 expression in HaCaT cells with transfection of sh-NC or sh-IGF2BP2. (c) CCK-8 assay for proliferation of HaCaT cells transfected with sh-NC or sh-IGF2BP2. (d) Wound healing analysis for migration of HaCaT cells with transfection of sh-NC or sh-IGF2BP2. (e) ELISA for VEGF level in HaCaT cells transfected with sh-NC or sh-IGF2BP2. (f) Tube formation assay for angiogenesis of HUVEC cells co-cultured with HaCaT cells transfected with sh-NC or sh-IGF2BP2. *p < 0.05, **p < 0.01, ***p < 0.001

To explore the role of IGF2BP2 in keratinocyte processes during wound healing, IGF2BP2 expression was knocked down in HaCaT cells using sh-IGF2BP2. As shown in Figure 2(a,b) and Supplementary Figure S1a, IGF2BP2 level was significantly decreased in HaCaT cells after transfection of sh-IGF2BP2 compared with sh-NC or non-transfected control group. Moreover, IGF2BP2 downregulation obviously reduced proliferation of HaCaT cells (Figure 2(c)). Additionally, IGF2BP2 knockdown markedly inhibited the migration of HaCaT cells (Figure 2(d)). Furthermore, angiogenesis was investigated by VEGF secretion and tube formation. Silencing IGF2BP2 significantly repressed VEGF secretion level in HaCaT cells (Figure 2(e)). In addition, by co-culturing HaCaT and HUVEC cells, tube-formation ability of HUVEC cells was significantly suppressed in sh-IGF2BP2 group (Figure 2(f)). These results showed IGF2BP2 downregulation repressed HaCaT cell proliferation, migration, and angiogenesis.

### IGF2BP2 knockdown reduces HPSE stability

**Figure 3.**
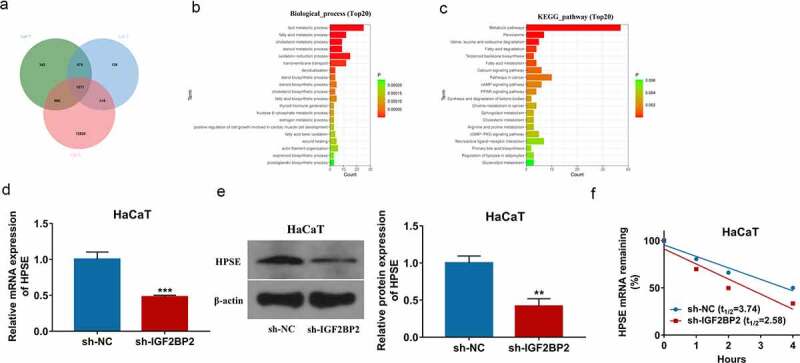
The regulation of IGF2BP2 on HPSE. (a) The overlapping targets of differentially expressed genes at day 7 (list 4), or day 14 (list 5) from GSE113081 dataset, and IGF2BP2-related genes from starBase database. (b) The biological process of GO assays of the overlapping targets using KOBAS. (c) KEGG pathway analysis of the overlapping targeting using KOBAS. (d and e) qRT-PCR and Western blotting for HPSE levels in HaCaT cells with transfection of sh-NC or sh-IGF2BP2. (f) The levels of HPSE mRNA after treatment with Actinomycin D for different times in HaCaT cells transfected with sh-NC or sh-IGF2BP2. **p < 0.01, ***p < 0.001

To explore the downstream targets of IGF2BP2 in keratinocyte processes during wound healing, the potential targets of IGF2BP2 were predicted by starBase database. By matching these predicted targets (list 6) and the two groups of differentially expressed genes in wounds on day 7 (list 4) and day 14 (list 5) from GSE113081 dataset, total of 1571 overlapping targets were obtained (Figure 3(a), Supplementary Table 2). Moreover, the 1571 overlapping targets were analyzed by GO annotations and KEGG enrichment analyses via KOBAS tool. As shown in Figures 3(b,c), those overlapping targets were involved in multiple functions in biological process and diverse pathways. Of note, the wound healing was present in the enrichment results (Supplementary Table 3). According to the GO annotations, HPSE was one of the important targets involved in the wound healing. Hence, we hypothesized IGF2BP2 might regulate wound healing through interacting with HPSE. Furthermore, IGF2BP2 downregulation significantly decreased HPSE expression in HaCaT cells (Figure 3(d,e) and Supplementary FigureS1b). In addition, Actinomycin D assay showed IGF2BP2 knockdown obviously decreased HPSE stability in the presence of Actinomycin D (figure 3(f)). These results indicated IGF2BP2 downregulation decreased HPSE stability in HaCaT cells.

### IGF2BP2 can bind with 3ʹ UTR of HPSE

**Figure 4.**
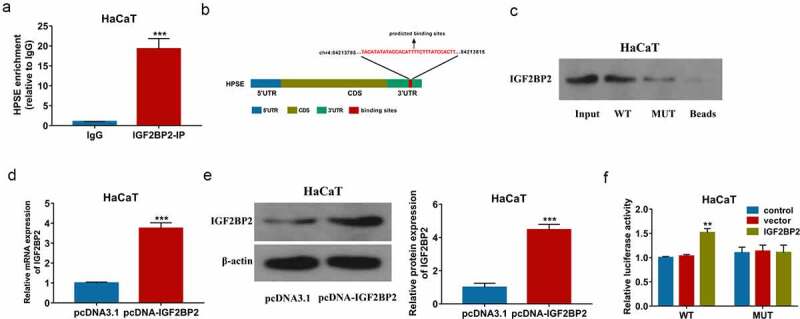
The interaction between IGF2BP2 and HPSE. (a) Enrichment of IGF2BP2 on HPSE by RIP assay. (b) The binding sites of IGF2BP2 on HPSE by starBase. (c) Enrichment of IGF2BP2 on HPSE by pull-down assay. (d and e) IGF2BP2 mRNA and protein levels in HaCaT cells transfected with pcDNA3.1 or pcDNA-IGF2BP2 using qRT-PCR and Western blotting. (f) Luciferase activity of WT-HPSE or MUT-HPSE in HaCaT cells transfected with pcDNA3.1 or pcDNA-IGF2BP2. **p < 0.01, ***p < 0.001

To study the interaction between IGF2BP2 and HPSE, the RIP assay was first performed in HaCaT cells. As displayed in Figure 4(a), abundant of HPSE was enriched by IGF2BP2-IP. The binding sites between IGF2BP2 and HPSE 3 ‘UTR were predicted by starBase, and exhibited in Figure 4(b). Furthermore, pull-down assay was conducted using WT or MUT HPSE probe. Lots of IGF2BP2 were enriched by WT HPSE probe, while this effect was clearly decreased by use of MUT HPSE probe (Figure 4(c)). In addition, the WT and MUT HPSE luciferase reporter vectors were constructed, and transfected in HaCaT cells. The transfection of pcDNA-IGF2BP2 obviously upregulated IGF2BP2 levels (Figure 4(d,e) and Supplementary Figure S1c). Moreover, IGF2BP2 upregulation significantly increased the luciferase activity of WT HPSE luciferase reporter vectors, but this effect was lost in the MUT group (figure 4(f)). These results suggested IGF2BP2 could bind with 3ʹ UTR of HPSE to increase HPSE stability in HaCaT cells.

### HPSE overexpression reverses the influences of IGF2BP2 downregulation on proliferation, migration, and angiogenesis

**Figure 5.**
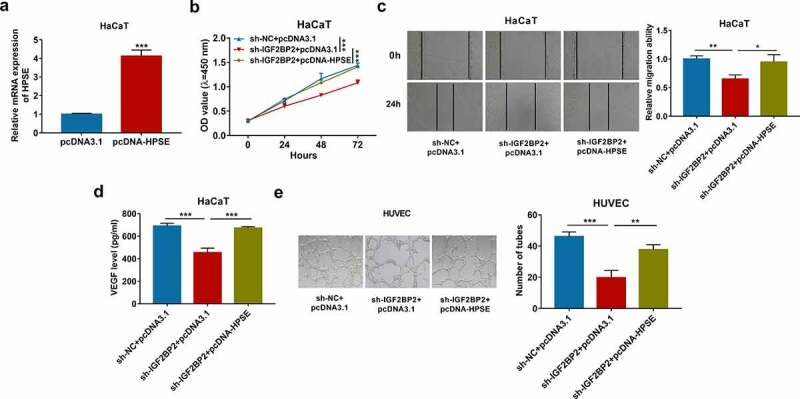
The effect of HPSE on IGF2BP2-mediated proliferation, migration and angiogenesis of HaCaT cells. (a) qRT-PCR for HPSE expression in HaCaT cells transfected with pcDNA3.1 or pcDNA-HPSE. (b) CCK-8 assay for proliferation of HaCaT cells transfected with sh-NC + pcDNA3.1, sh-IGF2BP2 + pcDNA3.1 or pcDNA-HPSE. (c) Wound healing analysis for migration of HaCaT cells with transfection of sh-NC + pcDNA3.1, sh-IGF2BP2 + pcDNA3.1 or pcDNA-HPSE. (d) ELISA for VEGF level in HaCaT cells transfected with sh-NC + pcDNA3.1, sh-IGF2BP2 + pcDNA3.1 or pcDNA-HPSE. (e) Tube formation assay for angiogenesis of HUVEC cells co-cultured with HaCaT cells with transfection of sh-NC + pcDNA3.1, sh-IGF2BP2 + pcDNA3.1 or pcDNA-HPSE. *p < 0.05, **p < 0.01, ***p < 0.001

To explore whether HPSE was required for IGF2BP2-mediated keratinocyte processes during wound healing, HaCaT cells were transfected with sh-NC + pcDNA3.1, sh-IGF2BP2 + pcDNA3.1 or pcDNA-HPSE. HPSE mRNA level was obviously elevated because of transfection of pcDNA-HPSE in HaCaT cells (Figure 5(a) and Supplementary Figure S1d). Moreover, HPSE overexpression mitigated silencing IGF2BP2-mediated reduction of HaCaT cell proliferation (Figure 5(b)). Furthermore, HPSE upregulation attenuated knockdown of IGF2BP2-meidated inhibition of migration of HaCaT cells (Figure 5(c)). In addition, increased HPSE abolished downregulation of IGF2BP2-mediated suppression of VEGF secretion and tube formation (Figure 5(d) and (e)). These results suggested IGF2BP2 knockdown constrained HaCaT cell proliferation, migration and angiogenesis by decreasing HPSE level.

## Discussion

The skin disruption can result in loss of the essential functions, so that improvement of wound healing is important in skin wound [26]. In this study, we firstly validated IGF2BP2 downregulation constrained keratinocyte proliferation, migration and angiogenesis, and found this effect was realized by regulating HPSE stability.

Previous studies showed adiponectin and Cysteine-rich angiogenic inducer 61 could promote skin wound healing through increasing keratinocyte proliferation and migration [27,28]. Moreover, the peptide-modified chitosan hydrogels and matrix metalloproteinase 13 could promote skin wound healing by enhancing angiogenesis [29,30]. These reports indicated the importance of keratinocyte proliferation, migration, and angiogenesis in skin wound healing. In our study, we found IGF2BP2 expression was increased in mice during wound healing, which was also consistent with a previous report [10], and the GSE28914 dataset in human (logFC = 0.669, p = 0.008). Besides, high homology of IGF2BP2 suggested the similar function in mouse and human. Wu *et a*l. reported IG2BP2, as a target of let-7b, increased keratinocyte migration in skin wound healing [10]. Consistent with the above report, this study also identified the pro-migratory function of IGF2BP2 in keratinocytes. Furthermore, IGF2BP2 exhibited pro-proliferation role in dental pulp cells and colorectal cancer cells [31,32]. Here, we firstly validated IGF2BP2 knockdown repressed keratinocyte proliferation. In addition, IGF2BP2 had a promoting effect on angiogenesis in lung cancer and leiomyoma cells [9,33]. VEGF is a key pro-angiogenic factor in skin wound healing [34,35]. Our study found IGF2BP2 silence attenuated angiogenesis by decreasing VEGF secretion and tube formation. These all suggested the effect of IGF2BP2 on keratinocyte processes, indicating IGF2BP2 might act as a promising targeting for wound healing treatment.

Next, we analyzed the downstream targets of IGF2BP2 and found HPSE might be a crucial target related to wound healing. Here, we firstly confirmed IGF2BP could bind with HPSE 3ʹUTR to increase its stability in keratinocytes. HPSE functioned as important upstream gene of VEGF, which increased the bioavailability of VEGF to promote angiogenesis [36,37]. Moreover, accumulated evidence suggested HPSE could increase cell proliferation, migration, and angiogenesis in different cell lines, including hepatocytes, pancreatic cancer cells, and cervical cancer cells [13,14,38]. In agreement with these previous reports, our study firstly confirmed the promoting effect of HPSE on keratinocyte proliferation, migration, and angiogenesis by overexpressing HPSE level under IGF2BP2 knockdown condition. Furthermore, we found HPSE overexpression mitigated the suppressive function of silencing IGF2BP2, indicating IGF2BP2 regulated keratinocyte processes through modulating HPSE stability. However, there were some limitations in the present work. First, the in vivo data were absent, so that the expression pattern and function of IGF2BP2 in vivo should be explored in future. Moreover, the HaCaT cells were different from primary keratinocytes. Hence, the further experiments using primary keratinocytes should be performed for better understand the mechanism of keratinocyte processes.

## Conclusion

Our study exhibits that IGF2BP2 knockdown repressed keratinocyte proliferation, migration, and angiogenesis by decreasing HPSE stability. This research indicates IGF2BP2 might serve as a new invention target for dealing with impaired wound healing in clinic, such as diabetes, vascular disease, and aging.

## Supplementary Material

Supplemental Material

Supplemental Material

## Highlights

1. The m6A-related IGF2BP2 is upregulated in wound healing.

2. IGF2BP2 knockdown inhibits proliferation, migration, and angiogenesis of HaCaT cells.

3. IGF2BP2 downregulation decreases HPSE expression.

4. IGF2BP2 interacts with HPSE by binding with the 3ʹ UTR.

5. HPSE overexpression reverses the influence of IGF2BP2 silence on HaCaT cell proliferation, migration, and angiogenesis.

## Abbreviations

m6A: N6-methyladenosine; IGF2BP2: Insulin-like growth factor 2 mRNA binding protein 2; HPSE: heparanase; GEO: Gene Expression Omnibus; FC: fold change; VEGF: vascular endothelial growth factor; GO: Gene Ontology; KEGG: Kyoto Encyclopedia of Genes and Genomes; DMEM: Dulbecco’s Modified Eagle Medium; CCK-8: cell counting kit-8; ELISA: enzyme linked immunosorbent assay; qRT-PCR: quantitative reverse transcription polymerase chain reaction; RIP: RNA immunoprecipitation; WT: wild-type; MUT: mutant; UTR: untranslated region
