# Supplementary material for: Insulin-like growth factor 2 mRNA binding protein 2 regulates proliferation, migration, and angiogenesis of keratinocytes by modulating heparanase stability
Source: Bioengineered. 2021 Dec 7;12(2):11267–76. doi: 10.1080/21655979.2021.2002495 (PMC8810085; doi:10.1080/21655979.2021.2002495)
Supplement: Supplemental Material [file KBIE_A_2002495_SM3490.zip › supplementary/Supplementary legend.docx]

**Supplementary Figure 1. The expression of IGF2BP2 and HPSE mRNA after the indicated transfection.** (A and B) qRT-PCR assay for IGF2BP2 and HPSE mRNA levels in HaCaT cells transfection with sh-NC or sh-IGF2BP2. (C) qRT-PCR assay for IGF2BP2 mRNA expression in HaCaT cells transfection with pcDNA3.1 or pcDNA-IGF2BP2. (D) qRT-PCR assay for HPSE mRNA expression in HaCaT cells transfection with pcDNA3.1 or pcDNA-HPSE. Control: non-transfected group. **p* < 0.05.

**Supplementary Table 1:** The differentially expressed genes in wounds on day 7 and 14 after wounding according to the GSE113081 dataset.

**Supplementary Table 2:** The overlapping targets, including the differentially expressed genes in wounds on day 7 vs 0 and day 14 vs 0 from GSE113081 dataset, and the predicted targets of IGF2BP2 by starBase database, were screened using jvenn tool.

The 1571 overlapping targets in Supplementary Table 2 were analyzed by GO annotations and KEGG enrichment analyses via KOBAS tool

**Supplementary Table 3:** Those overlapping targets in Supplementary Table 2 were involved in multiple functions in biological process and diverse pathways.
